# Contemporary Zika Virus Isolates Induce More dsRNA and Produce More Negative-Strand Intermediate in Human Astrocytoma Cells

**DOI:** 10.3390/v10120728

**Published:** 2018-12-19

**Authors:** Trisha R. Barnard, Maaran M. Rajah, Selena M. Sagan

**Affiliations:** 1 Department of Microbiology and Immunology, McGill University, Montreal, QC H3A 2B4, Canada; trisha.barnard@mail.mcgill.ca (T.R.B.); michael.rajah@mail.mcgill.ca (M.M.R.); 2 Department of Biochemistry, McGill University, Montreal, QC H3A 2B4, Canada

**Keywords:** Zika virus, flavivirus, astrocytomas, dsRNA, viral fitness

## Abstract

The recent emergence and rapid geographic expansion of Zika virus (ZIKV) poses a significant challenge for public health. Although historically causing only mild febrile illness, recent ZIKV outbreaks have been associated with more severe neurological complications, such as Guillain-Barré syndrome and fetal microcephaly. Here we demonstrate that two contemporary (2015) ZIKV isolates from Puerto Rico and Brazil may have increased replicative fitness in human astrocytoma cells. Over a single infectious cycle, the Brazilian isolate replicates to higher titers and induces more severe cytopathic effects in human astrocytoma cells than the historical African reference strain or an early Asian lineage isolate. In addition, both contemporary isolates induce significantly more double-stranded RNA in infected astrocytoma cells, despite similar numbers of infected cells across isolates. Moreover, when we quantified positive- and negative-strand viral RNA, we found that the Asian lineage isolates displayed substantially more negative-strand replicative intermediates than the African lineage isolate in human astrocytoma cells. However, over multiple rounds of infection, the contemporary ZIKV isolates appear to be impaired in cell spread, infecting a lower proportion of cells at a low MOI despite replicating to similar or higher titers. Taken together, our data suggests that contemporary ZIKV isolates may have evolved mechanisms that allow them to replicate with increased efficiency in certain cell types, thereby highlighting the importance of cell-intrinsic factors in studies of viral replicative fitness.

## 1. Introduction

The recent emergence and rapid geographic expansion of the mosquito-borne Zika Virus (ZIKV) poses a significant burden on the global health infrastructure [1]. The virus was initially isolated in 1947 from sentinel rhesus macaques in the Zika forest region of Uganda [2]. ZIKV circulated throughout Africa as well as in Southeast Asia over the latter half of the twentieth century, where it caused sporadic infections resulting in mild febrile illness [3,4]. The first major transmission of ZIKV outside of its endemic zone occurred in 2007, where 73% of the population of Yap Island, Federated States of Micronesia contracted the virus within a four-month period [4,5]. However, the clinical manifestations of ZIKV infection during the Yap Island epidemic were relatively similar to historical descriptions; resulting in mild, self-limiting, febrile illness characterized by rash, arthralgia, conjunctivitis, and headaches [3,5]. Interestingly, the continued geographic expansion of the ZIKV epidemic coincided with reports of novel neurological pathogenesis, starting with the 2013 French Polynesian epidemic, which saw a drastic increase in reports of Guillain-Barré syndrome [6].

Following the French Polynesian outbreak, the trans-Pacific transmission of ZIKV resulted in several epidemics throughout the Americas, the most salient being the Brazilian epidemic (2014–2016), which coincided with a 20-fold increase in incidence of congenital malformations, including fetal microcephaly from 2014 to 2015 [7]. Notably, ZIKV outbreaks in South America were also associated with neurological complications, and several investigations now suggest a connection between ZIKV infection and the development of Guillain-Barré syndrome, as well as congenital birth defects, including fetal microcephaly [4,8,9,10,11,12]. In addition to epidemiological factors, such as the increased mobility of infected individuals and the immunological naivety of recently-afflicted human populations, novel genomic polymorphisms acquired by contemporary outbreak strains likely contributed to ZIKV pathogenicity and dissemination in recent outbreaks [13]. Studies conducted in human neurospheres, cerebral organoids, and primary astrocytes, as well as in murine models of infection, have demonstrated differences in neurotropism, pathogenicity, and the antiviral responses between Asian and African lineage isolates [14,15,16,17]. Furthermore, a recent investigation uncovered a single nucleotide substitution in the prM region of the viral polyprotein that increases ZIKV infectivity in human and mouse neural progenitor cells and leads to significant fetal microcephaly in mice, resulting in greater mortality of neonatal mice [18]. Additionally, another polymorphism found in the NS1 region of contemporary ZIKV isolates results in increased NS1 antigenemia in mice, enhanced infectivity in *Aedes* mosquitoes, and reduced induction of antiviral signaling in human cells [19,20]. Thus, characterizing the difference in viral replicative fitness between the contemporary epidemic strains to the pre-epidemic strains could help to provide an evolutionary context for the emergence and rapid dissemination of ZIKV in the recent outbreaks.

Herein, we sought to compare viral replicative fitness by investigating viral growth kinetics, cytopathicity, and viral RNA accumulation of contemporary epidemic (2015–2016) and pre-epidemic ZIKV isolates in two cell culture models of ZIKV infection. First, we chose to use the A549 human lung epithelial carcinoma cells in order to contextualize our results within the literature, since A549 cells are widely used in ZIKV research [21,22,23]. Although A549 cells were reported to be a resilient model of ZIKV infection [21], the lung is not a target of ZIKV infection in vivo [24]. In contrast, several studies have shown that astrocytes are a primary target of ZIKV infection in vivo [16,25,26], and a recent study demonstrated that the U-251 MG human astrocytoma cell line is more permissive to ZIKV infection than A549 cells [27]. Therefore, we chose to use the U-251 MG cell line because an astrocyte-derived cell type may be a more relevant model for ZIKV-induced neuropathology and be better able to distinguish differences between ZIKV isolates. We found that contemporary ZIKV isolates (from Puerto Rico and Brazil) appear to have an increase in viral replicative fitness in astrocytoma cells over a single infectious cycle, with significantly more double-stranded RNA (dsRNA)-positive cells when compared to pre-epidemic isolates, despite similar numbers of infected cells. Moreover, when we investigated viral RNA accumulation, we found that the Asian lineage isolates had a substantially greater proportion of negative-strand intermediates than the African lineage isolate in both A549 and astrocytoma cells. However, over multiple rounds of infection, the contemporary ZIKV isolates appear to be impaired in cell spread, infecting a lower proportion of cells, despite the production of similar or higher titers. Our results suggest that the contemporary ZIKV isolates may have evolved mechanisms that allow them to replicate with increased efficiency in certain cell types and highlight the importance of cell-intrinsic factors in studies of viral replicative fitness.

## 2. Materials and Methods

### 2.1. Phylogenetic Analysis

Translated amino acid sequences of 50 ZIKV polyproteins (Table S1) were aligned using ClustalW [28]. Trees were constructed by neighbor joining of pairwise amino acid distances with the program MEGA7 (according to the distance scale provided) [29]. Bootstrap resampling was used to determine robustness of branches; values of ≥50% (from 1000 replicates) were used.

### 2.2. Cells and Viruses

African green monkey kidney (Vero) cells, human embryonic kidney (293T) cells, human lung carcinoma (A549) cells, and human astrocytoma (U-251 MG) cells were kindly provided by Martin J. Richer (McGill University, Montreal, QC, Canada), Connie Krawczyk (McGill University, Montreal, QC, Canada), Russell Jones (McGill University, Montreal, QC, Canada), and Anne Gatignol (Lady Davis Research Institute, Montreal, QC, Canada), respectively. All cells were maintained in Dulbecco’s modified Eagle’s medium (DMEM) supplemented with 10% fetal bovine serum (FBS), 1% nonessential amino acids, 1% l-glutamine, and 1% penicillin/streptomycin at 37 °C/5% CO_2_.

An infectious cDNA of ZIKV strain MR-766 (ZIKV^AF^; Genbank accession: HQ234498.1) was kindly provided by Matthew Evans (Mount Sinai, NY, USA) [30]. ZIKV^AF^ viral stocks were generated by transfection of 293T cells with the infectious cDNA using Lipofectamine 2000 (Life Technologies, Thermo Fisher Scientific, Waltham, MA, USA) followed by a single passage in Vero cells. ZIKV isolate PLCal_ZV (ZIKV^CDN^; Genbank accession: KF99378) was generously provided by David Safronetz (National Microbiology Laboratories, Winnipeg, MB, Canada) [31]. Isolates PRVABC59 (ZIKV^PR^; Genbank accession: KU501215) and HS-2015-BA-01 (ZIKV^BR^; Genbank accession: KX520666) were provided by Tom Hobman (University of Alberta, Edmonton, AB, Canada) and Mauro Teixeira (Universidade Federal de Minas Gerais, Belo Horizonte, Brazil), respectively. The passage history of each ZIKV isolate is described in Table S2.

### 2.3. ZIKV Infections

A549 and U-251 MG cells were seeded at a density of 4 × 10^4^ cells per well in 12-well plates the day before infection. Virus was diluted to the indicated MOI in Eagle’s minimum essential medium (EMEM; Wisent Inc., St-Bruno, QC, Canada) and was allowed to bind to cells for 1 h at 37 °C/5% CO_2_, after which the inoculum was removed, cells were washed with PBS, and media was replaced with fresh media containing 15 mM HEPES (Wisent Inc.) and 2% FBS. At the specified time points, the supernatant was collected and clarified by centrifugation at 4 °C for 10 min at 3000× *g*, and stored at −80 °C prior to titration.

Viral titers were determined by plaque forming unit (PFU) assay on Vero cells. Briefly, 500 μL of 10-fold serial dilutions were incubated for 2 h on Vero cell monolayers in 12-well plates. The virus inoculum was removed, and the cells were overlaid with DMEM containing 1.2% Carboxymethyl cellulose (Sigma-Aldrich, Oakville, ON, Canada), 2% FBS, and 1% penicillin/streptomycin. Four days post-infection, cells were fixed with 5% formaldehyde and stained with 0.1% crystal violet (Sigma-Aldrich) to visualize plaques.

### 2.4. Cell Viability

Cell viability was monitored using a modified 3-(4,5-dimethylthiazol-2-yl)-2,5- diphenyltetrazolium bromide (MTT) assay similar to what has been previously described [32]. Briefly, cells were plated at density of 2000 cells per well in flat-bottom 96-well plates and allowed to adhere overnight. Ten wells per strain were infected with 100 µL of ZIKV diluted to the indicated MOI. At the specified time points, 10 µL of 5 mg/mL MTT salt (Sigma-Aldrich) in EMEM solution was added to each well and incubated for 4 h at 37 °C, after which 100 µL of 10% SDS in 0.01 M HCl was added per well and the plates were incubated overnight at 37 °C. Absorbance at 550 nm with a reference wavelength of 650 nm was read on a Spark 10M plate reader (Tecan, Männedorf, Switzerland). The average absorbance of 10 wells was used and viability experiments were carried out in triplicate. The data was expressed in % Cytopathicity, which was defined as: % Cytopathicity = 100% − ((Uninfected Absorbance − Infected Absorbance)/(Uninfected Absorbance) × 100%).

### 2.5. Flow Cytometry

A549 and U-251 MG cells were seeded at a density of 1 × 10^6^ cells per 15-cm^2^ plate the day before infection. Cells were infected with ZIKV at indicated MOI as described above and harvested at the indicated time points. Prior to fixation, cells were stained with Fixable Viability Dye eFluor 780 (Thermo Fisher Scientific, Waltham, MA, USA) according to the manufacturer’s instructions. Cells were fixed using Cytofix/Cytoperm (BD Bioscience, Mississauga, ON, Canada) for 20 min and then stained with anti-J2 dsRNA (Scicons, Szirák, Hungary) diluted 1:1000, anti-4G2 (Millipore, Etobicoke, ON, Canada) diluted 1:200, or anti-cleaved Caspase-3 (Cell Signaling, Danvers, MA, USA) diluted 1:800 in PermWash (BD Bioscience) for 1 h followed by secondary staining with goat anti-mouse Alexafluor 488 or goat anti-rabbit Alexafluor A546 (Thermo Fisher Scientific, Waltham, MA, USA) diluted 1:300 for dsRNA or 1:500 for 4G2 and Caspase-3 in PermWash. Data were acquired on an LSRFortessa Analyzer (BD Bioscience) with BD FACSDiva software. Data analysis was performed using FlowJo software version 10.5 (BD Bioscience, Mississauga, ON, Canada). Debris and doublets were excluded from the analysis using forward-scatter width discrimination, and the percentage of dsRNA-, 4G2-, or Caspase-3-positive cells was determined by comparison to mock-infected cells.

### 2.6. Immunofluorescence Microscopy

A549 and U-251 MG cells were seeded at a density of 2 × 10^4^ cells per well in eight-well chamber slides the day before infection. Cells were infected with ZIKV at indicated MOI as described above and harvested at the indicated time points. Cells were fixed in 4% formaldehyde in 1× PBS, washed three times in PBS, and blocked with 0.3% Triton X-100 and 5% FBS in PBS for 1 h at room temperature. Samples were incubated with anti-J2 dsRNA (Scicons) diluted 1:1000 in PBS containing 1% BSA and 0.3% Triton X-100 at room temperature for 1 h followed by washing three times in PBS. Samples were then incubated with goat anti-mouse Alexafluor 488 (Thermo Fisher Scientific) diluted 1:300 in PBS containing 1% BSA and 0.3% Triton X-100 followed by three PBS washes before mounting with VectaShield containing DAPI (Cedarlane, Burlington, ON, Canada). Z-stack images were acquired using a Zeiss AxioObserver inverted microscope with a 63× oil objective. The number of dsRNA foci per cell was quantified using Imaris version 9.1.2 (Bitplane Inc., South Windsor, CT, USA). Foci of size 0.3 × 0.3 × 4 μm above quality 17.0 were automatically detected using the spot detection function and manually verified for at least 100 cells per condition.

### 2.7. Quantitative Reverse-Transcription PCR

Total RNA was extracted from cells using Trizol reagent (Thermo Fisher Scientific) following the manufacturer’s protocol. Quantification of positive- and negative-strand intracellular viral RNA was determined by quantitative reverse-transcription PCR (qRT-PCR) on a Bio-Rad CFX96 Touch Real-Time System using the iTaQ Universal Probe One-Step Kit (Bio-Rad, Mississauga, ON, Canada) with 0.5 μL of PrimePCR GAPDH primers and probe (HEX, Bio-Rad) per reaction. The primers used to detect ZIKV RNA were as follows: forward, 5′-CCG CTG CCC AAC ACA AG-3′; reverse, 5′-CCA CTA ACG TTC TTT TGC AGA CAT-3′; probe, 5′-/56-FAM/AGC CTA CCT/ZEN/TGA CAA GCA ATC AGA CAC TCA A/3IABkFQ/-3′ [33]. A strand-specific reverse transcription (RT) reaction was carried out by addition of either the forward or the reverse primer during RT at 50 °C for 10 min, after which the other ZIKV primer and ZIKV probe was added to the reaction. The cycling conditions were: 95 °C for 3 min, then 45 cycles of 95 °C for 5 s and 60 °C for 60 s. The amount of viral RNA was normalized to GAPDH using the ΔCt method, and compared to a standard curve of log_10_ (PFU equivalents) which was extracted from cell culture supernatants [8], using the NucleoSpin RNA virus kit (Macherey-Nagel, Bethlehem, PA, USA) following the manufacturer’s instructions. The relative amount of positive- and negative-strand viral RNA was calculated using the 2^(−ΔΔCT)^ method using GAPDH as the internal control after normalization to the relative PCR efficiencies of the different ZIKV isolates.

### 2.8. Statistical Analysis

All statistical analyses were performed using Prism 6 software (GraphPad, San Diego, CA, USA). At each time point, viral titers from growth curves, % cytopathicity, % infected cells, or viral RNA were compared using a one-way analysis of variance (ANOVA) with Tukey’s multiple-comparison test.

## 3. Results

### 3.1. Phylogenetic and Amino Acid Variance across ZIKV Isolates Selected for Comparative Analyses

Phylogenetic analyses demonstrate that ZIKV can be divided into two main lineages: African and Asian (Figure S1) [34,35]. The African lineage has caused sporadic infections over the last century, typically resulting in mild, febrile illness [1]. All of the strains identified in the 2014–2016 epidemic are of the Asian lineage and are more closely related to the H/PF/2013 French Polynesia strains than the FSM/2007 Micronesia (Yap Island) strain, suggesting that these sub-lineages may have evolved independently from a common ancestor, anchored by the P6-740 strain (Malaysia, 1966) (Figure S1) [34]. Notably, ZIKV outbreaks in French Polynesia and South America were the first to be associated with neurological symptoms, including Guillain-Barré syndrome and fetal microcephaly [4,9,36]. As such, we sought to perform a comparative analysis of historical and contemporary ZIKV isolates to study the impact of genetic polymorphisms on viral replicative fitness and cytopathicity in cell culture. We chose a panel of ZIKV isolates including: Uganda 1947 (MR766, ZIKV^AF^); an early Asian lineage strain, isolated from a Canadian traveller whom returned from Thailand viremic in 2013 (PLCal_ZV, ZIKV^CDN^); and two isolates from the 2015 outbreaks in Puerto Rico (PRVABC59, ZIKV^PR^) and Brazil (HS-2015-BA-01, ZIKV^BR^). Notably, ZIKV^AF^ was mouse passaged and all of the other viral isolates were passaged through Vero cells or a combination of Vero and C6/36 mosquito cells (Table S2). A detailed amino acid sequence comparison of these strains is available in File S1. Comparison of the overall amino acid composition across strains demonstrates that the maximum amino acid differences are between the ZIKV^AF^ and ZIKV^PR^ isolates (Table S3). Polymorphisms are located throughout the viral polyprotein; however, several regions (prM, E, NS2A, NS4B, and NS5 proteins) have a higher accumulation of amino acid polymorphisms, particularly between the African and Asian lineages (File S1).

### 3.2. Zika Virus Isolates Display Unique Plaque Morphology and Different Growth Kinetics in Cell Culture

**Figure 1:**
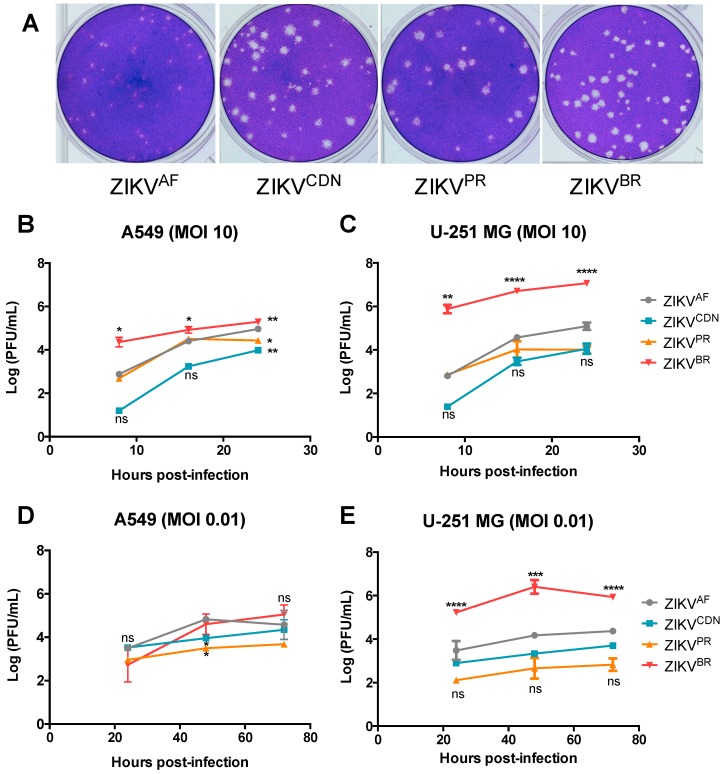
ZIKV isolates demonstrate unique plaque morphology and different growth kinetics in A549 and U-251 MG cell lines. (**A**) Representative images of Vero cell plaque assays of the indicated ZIKV isolates. (**B**–**E**) Cell culture supernatants were collected at the indicated time points and viral titer was determined by plaque assay. (**B**) A549 and (**C**) U-251 MG cells were infected with ZIKV at MOI = 10. (**D**) A549, and (**E**) U-251 MG cells were infected with ZIKV at MOI = 0.01. Values represent mean ± SD of at least three independent experiments. Asterisks indicate significant differences in viral titer relative to ZIKV^AF^: * *p* < 0.05, ** *p* < 0.01, *** *p* < 0.001, **** *p* < 0.0001.

While preparing viral stocks we noticed differences in plaque morphology on Vero cells between the four ZIKV isolates (Figure 1). Plaques from the historical ZIKV^AF^ were smaller than plaques from the Asian lineage isolates and were more uniform in size (Figure 1A). Of the Asian lineage isolates, both ZIKV^CDN^ and ZIKV^PR^ produced plaques with indefinite borders, whereas the plaques from the ZIKV^BR^ had clearly defined edges (Figure 1A). In order to better characterize each viral isolate, we investigated strain-specific differences in growth kinetics, cytopathicity, infectivity, and viral RNA accumulation.

We first sought to determine the viral growth kinetics for each of the four ZIKV isolates (Figure 1B–E). Viral titers were assessed using both one-step and multi-step growth curves after infection at an MOI of 10 and 0.01, respectively (Figure 1B–E). At MOI of 10, ZIKV^BR^ grew to significantly higher titers at 8 h post-infection in both cell types, suggesting that ZIKV^BR^ has an advantage in terms of growth kinetics (Figure 1B,C). In A549 cells, the increased titer of ZIKV^BR^ was much less pronounced by 24 h post-infection, at which time the titers of the other isolates have nearly caught up to within 1-log of ZIKV^BR^ (Figure 1B). In contrast, in U-251 MG cells, ZIKV^BR^ continues to replicate to significantly higher titers than all other isolates at all time points post-infection, even when titers have begun to plateau by 24 h post-infection (Figure 1C). Interestingly, the ZIKV^BR^ titers are already increasing by 8 h post-infection, whereas titers for the other isolates do not increase until after 8 h post-infection based on titers at 0 h [37]. Overall, this suggests that ZIKV^BR^ replicates to higher titers in U-251 MG cells, with faster replication kinetics over a single infectious cycle.

To further investigate the differences in viral replication kinetics between isolates, we also performed a multi-step growth curve at MOI of 0.01 (Figure 1D,E). In A549 cells, although at 48 h post-infection ZIKV^CDN^ and ZIKV^PR^ have lower titers than ZIKV^AF^, there are no significant strain-dependent differences in viral titers at any other time point post-infection (Figure 1D). In contrast, in the U-251 MG cells, ZIKV^BR^ grew to significantly higher titers at all time points post-infection when compared to all other viral isolates (Figure 1E). There were no significant differences in viral titers between the remaining isolates in U-251 MG cells, although ZIKV^AF^ tended to have slightly higher titers than ZIKV^CDN^ and ZIKV^PR^ (Figure 1E). Overall, the data demonstrates that over multiple rounds of infection, ZIKV^BR^ appears to have a significant replicative advantage in U-251 MG cells, growing to higher titers than all other ZIKV isolates.

### 3.3. Cytopathic Effects Induced by ZIKV Isolates in Cell Culture Depends on MOI

**Figure 2:**
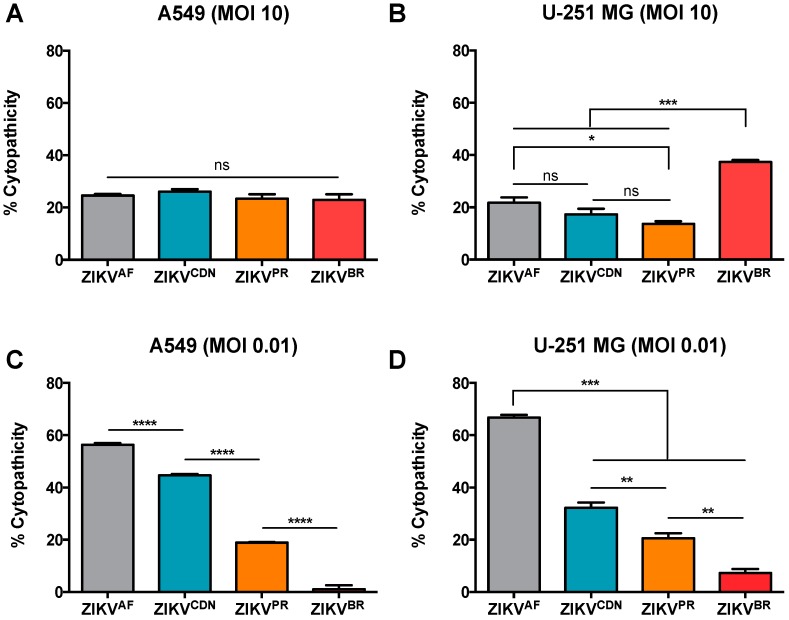
ZIKV isolates elicit different cytopathic effects in A549 and U-251 MG cell lines. (**A**) A549 and (**B**) U-251 MG cells were infected with ZIKV at MOI = 10 and cell viability was determined by MTT assay at 24 h post-infection. (**C**) A549 and (**D**) U-251 MG cells were infected with ZIKV at MOI = 0.01 and cell viability was determined by MTT assay 72 h post-infection. % Cytopathicity = 100% − ((Uninfected Absorbance − Infected Absorbance)/(Uninfected Absorbance) × 100%). Values represent the mean ± SEM of three independent experiments. Asterisks indicate significant differences in % cytopathicity: * *p* < 0.05, ** *p* < 0.01, *** *p* < 0.001, **** *p* < 0.0001.

We next wanted to determine whether there was a connection between viral particle production and the ability to induce cytopathic effects (CPE) in cell culture. Using an MTT assay to investigate strain-specific differences in cytopathicity, we observed that when cells were infected at MOI of 10, CPE induced by the four ZIKV isolates was consistent with the viral load (Figure 2). More specifically, in the A549 cells, all isolates induced similar CPEs with approximately 25% cytopathicity, which represents a 25% reduction in cell viability at 24 h post-infection (Figure 2A), with similar viral titers at this time point (Figure 1B). In contrast, in the U-251 MG cells, ZIKV^BR^ was significantly more cytopathic at the high MOI, inducing approximately 37% cytopathicity compared with an average of 18% cytopathicity at 24 h post-infection induced by the other ZIKV isolates (Figure 2B). Again, this finding is in good agreement with the relative viral titers at this time point (Figure 1C). To determine whether CPEs were apoptosis-driven or due to other forms of cell death, we quantified cleaved caspase-3 positive cells by flow cytometry at MOI of 10 in both cell types (Figure S2). In A549 cells, apoptosis appears to account for a small proportion of cell death with 3.5%, 1.9%, and 7.2% cleaved caspase-3-positive cells during ZIKV^AF^, ZIKV^CDN^, and ZIKV^PR^ infection, respectively; however, during ZIKV^BR^ infection approximately 47% of cells stained positive for cleaved caspase-3 (Figure S2A). In U251-MG cells, a similar pattern was observed with fewer cells staining positive for cleaved caspase-3 (between 0.6–1.43% for ZIKV^AF^, ZIKV^CDN^ and ZIKV^PR^), whereas approximately 17.1% of cells were cleaved caspase-3-positive during ZIKV^BR^ infection (Figure S2B). This suggests that, at least for ZIKV^AF^, ZIKV^CDN,^ and ZIKV^PR^, apoptosis is not the main driver of CPEs during ZIKV infection, and thus, other forms of cell death are likely to contribute to this phenotype. However, apoptosis appears to have a greater contribution to CPEs observed during infection with ZIKV^BR^. Taken together, this suggests that over a single infectious cycle, ZIKV^BR^ induces more CPEs in U-251 MG cells than the other ZIKV isolates, likely due to an increase in apoptosis. In addition, at least at an MOI of 10, CPEs appear to be directly correlated with the viral titer for all four ZIKV isolates.

Next, we determined the relative CPEs induced by the four ZIKV isolates over multiple rounds of infection at a low MOI. In contrast to the high MOI condition, where strain-specific differences in CPE were dependent on cell type, at low MOI, the strain-specific trend in CPE was consistent across cell lines (Figure 2C,D). At 72 h post-infection with MOI 0.01, ZIKV^BR^ induced the least CPEs in both A549 and U-251 MG cells with approximately 2% and 7% cytopathicity, respectively (Figure 2C,D), despite generating similar or higher titers at this time point (Figure 1D,E). ZIKV^PR^ and ZIKV^CDN^ induced intermediate CPE in both cell types (approximately 40% and 20% cytopathicity, respectively). However, ZIKV^AF^ induced the greatest CPE of all isolates at 72 h post-infection at MOI 0.01, with approximately 60% cytopathicity in both A549 and U-251 MG cells (Figure 2C,D). Taken together, these results suggest that although ZIKV^BR^ appears to have a replicative advantage that is more pronounced in astrocytoma cells, this advantage is likely due to increased viral replication in the initial infection rather than to increases over subsequent rounds of infection. As this could imply differences in cell spread across the ZIKV isolates, we next wanted to assess the percentage of infected cells across isolates.

### 3.4. Flow Cytometry Analysis Reveals Strain-Specific Differences in Viral Infectivity and Cell Spread

**Figure 3:**
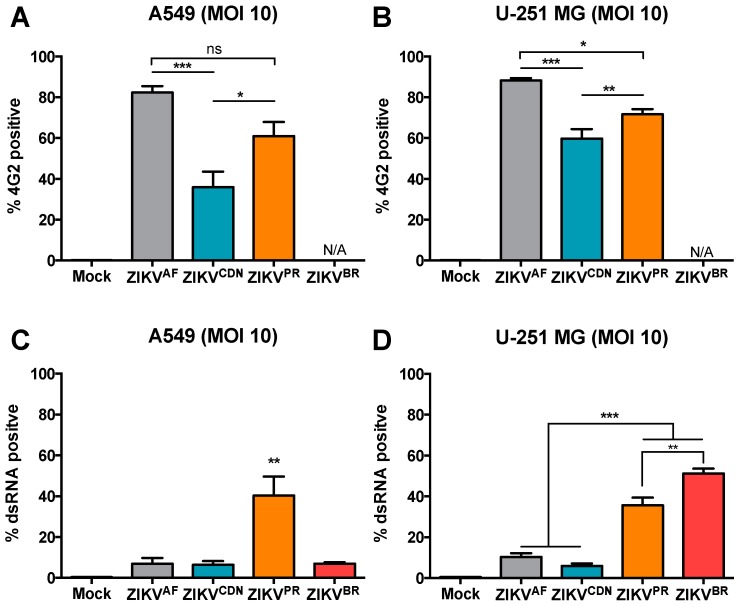
Contemporary ZIKV isolates induce more dsRNA than pre-epidemic isolates, despite similar numbers of infected cells. (**A**) A549 and (**B**) U-251 MG cells were infected with ZIKV at MOI = 10 and at 24 h post-infection cells were stained with the pan-flavivirus (4G2) antibody and the percentage of infected cells was determined by flow cytometry. (**C**) A549 and (**D**) U-251 MG cells were infected with ZIKV at MOI = 10 and 24 h post-infection the percentage of dsRNA-positive cells was determined by flow cytometry. The percentage of positive cells was determined by comparison to mock-infected cells. Values represent mean ± SEM of at least three independent experiments. Asterisks indicate significant differences in % infected cells: * *p* < 0.05, ** *p* < 0.01, *** *p* < 0.001.

**Figure 4:**
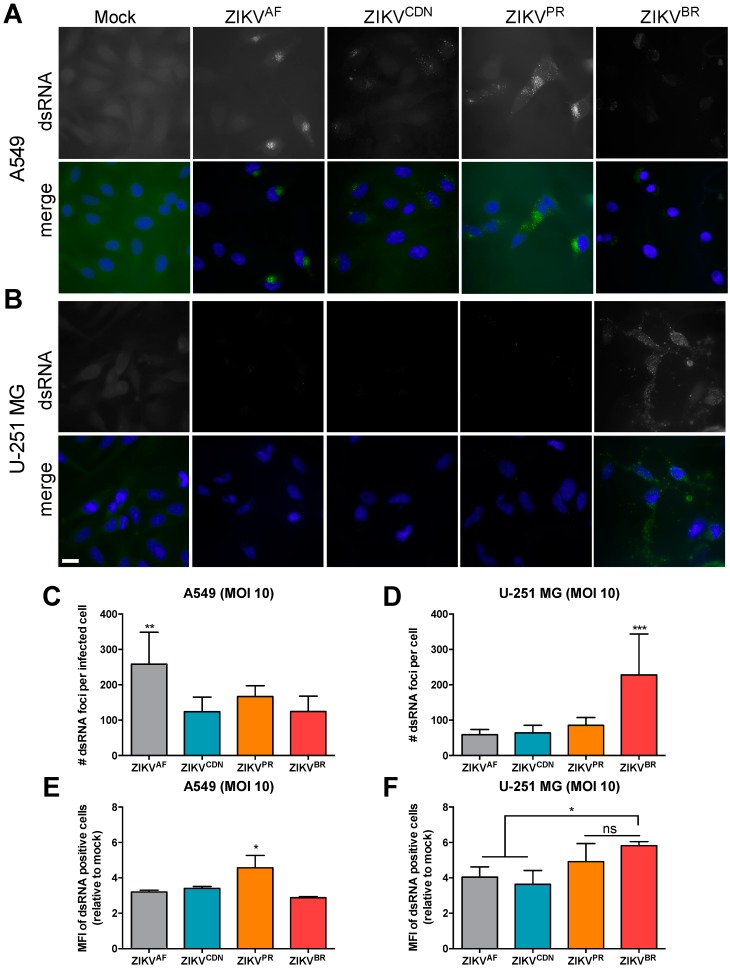
Isolate-specific differences are observed in number and fluorescence intensity of dsRNA foci in infected cell. (**A**) A549 and (**B**) U-251 MG cells were infected with ZIKV at MOI = 10 and 24 h post-infection dsRNA expression was analyzed by immunofluorescence microscopy. Scale bar, 20 μm. The number of dsRNA foci per cell in (**C**) A549 and (**D**) U-251 MG cells was quantified using Imaris software (>100 cells/condition). (**E**) A549 and (**F**) U-251 MG cells were infected with ZIKV at MOI = 10 and 24 h post-infection the mean fluorescence intensity (MFI) of dsRNA-positive cells was determined by flow cytometry. Values represent mean ± SEM of at least three independent experiments. Asterisks indicate significant differences: * *p* < 0.05, ** *p* < 0.01, *** *p* < 0.001.

Due to the discrepancies in CPEs elicited at different MOIs, we set out to determine the percent of ZIKV-infected cells at high and low MOI using flow cytometry (Figure 3 and Figure S3). We initially determined the % infected cells by staining for the envelope glycoprotein using a pan-flavivirus (4G2) antibody (Figure 3A,B). At MOI 10, ZIKV^CDN^ infected a lower proportion of A549 cells (36%) when compared to ZIKV^AF^ (82%) and ZIKV^PR^ (61%) at 24 h post-infection (Figure 3A). A similar trend was observed in the U251-MG cells, where ZIKV^AF^, ZIKV^CDN^, and ZIKV^PR^ infected 88%, 60%, and 72% of cells, respectively (Figure 3B). However, we were unable to detect ZIKV^BR^ using the envelope antibody in either cell line. Given that viral epitopes may have changed over the course of the viral evolutionary history, we stained for dsRNA as a marker for active viral replication, to ensure similar sensitivity of detection across all isolates. At MOI 10, we detected significantly more dsRNA in a higher proportion of A549 cells during ZIKV^PR^ infection (40%), whereas only approximately 7% of A549 cells were dsRNA-positive in the other three isolates (ZIKV^AF^, ZIKV^CDN^, and ZIKV^BR^) at 24 h post-infection (Figure 3C). In the U251-MG cells, the rate of infection was similar to that in A549 cells for ZIKV^AF^ and ZIKV^CDN^, with approximately 10% and 6% dsRNA-positive cells, respectively (Figure 4D). However, for both the contemporary isolates (ZIKV^PR^ and ZIKV^BR^), a significantly higher percentage of cells were dsRNA-positive in U-251-MG cells, with approximately 36% and 51% dsRNA-positive cells, respectively (Figure 3D). These results suggest that ZIKV^PR^ may induce more viral RNA replication (as indicated by dsRNA staining) than ZIKV^AF^ and ZIKV^CDN^, since all three isolates display a similar % of infected cells when staining for the viral envelope, but vastly different amounts of dsRNA-positive cells. This may also be true for ZIKV^BR^, which had a similar percentage of dsRNA-positive cells; however, we were not able to assess the % infected cells for this isolate using the envelope antibody. Taken together, these results suggest that over a single infectious cycle, ZIKV^PR^ infection results in a greater proportion of dsRNA-positive cells in both cell types, while ZIKV^BR^ selectively induces more dsRNA-positive cells in U-251 MG cells.

At the low MOI (0.01), the trend in % of dsRNA-positive cells closely mirrored the trend in cytopathicity (Figure 2 and Figure S3). At 72 h post-infection at MOI 0.01, ZIKV^AF^ has the highest % of dsRNA-positive cells in both the A549 and U-251-MG cells, with 30% and 15% dsRNA-positive cells, respectively (Figure S3A,B). All of the other ZIKV isolates had a lower % dsRNA-positive cells in both cell types (Figure S3A,B), consistent with the intermediate or low CPEs observed at this MOI (Figure 2C,D). However, dsRNA staining is likely an underestimation the % infected cells if the viral isolates do not induce high levels of dsRNA. Nonetheless, these results suggest that although ZIKV^BR^, and possibly also ZIKV^PR^, appear to have a replicative fitness advantage over a single infectious cycle that is more pronounced in astrocytoma cells, they appear to be impaired in cell spread when subjected to multiple rounds of infection at low MOI.

### 3.5. The Number of dsRNA Foci and Ratio of Negative- to Positive-Strand RNA Differ across African and Asian Lineage Isolates

We next wanted to determine whether the increase in the % of dsRNA-positive cells observed during ZIKV^PR^ and ZIKV^BR^ infection was due to an increased number of replication complexes induced per cell. Thus, we quantified the number of dsRNA foci by immunofluorescence microscopy and image analyses at MOI of 10 in A549 and U-251 MG cells (Figure 4A,B). In A549 cells, ZIKV^AF^ yielded the greatest number of dsRNA foci per infected cell, with on average >250 dsRNA foci/cell, while the Asian lineage isolates had, on average, approximately 124–166 dsRNA foci/cell (Figure 4C). In contrast, in the U-251 MG cells, the Brazilian isolate had the greatest number of dsRNA-positive foci, with >225 dsRNA-positive foci/cell, while all other isolates had <100 dsRNA foci/cell on average (Figure 4D). To further confirm these findings, we calculated the mean fluorescence intensity (MFI) of dsRNA-positive cells by flow cytometry (Figure 4E,F). The MFI revealed similar fluorescent intensities across ZIKV isolates in A549 cells, with the exception of ZIKV^PR^, which were significantly more intense in this cell type (Figure 4E). Moreover, in U-251 MG cells, the contemporary isolates displayed a higher MFI for dsRNA-positive cells, suggesting that there were greater amounts of dsRNA per cell (Figure 4F). Taken together, this suggests that although the ZIKV^AF^ isolate induced more dsRNA foci in A549 cells, they were on average of low fluorescence intensity. In contrast, in U-251 MG cells, ZIKV^BR^ produced substantially more dsRNA-positive foci, and together with ZIKV^PR^, the foci were brighter than those of the ZIKV^AF^ and ZIKV^CDN^ isolates. However, we cannot rule out the existence of a larger proportion of small dsRNA foci in these cells as our image analysis relied on a minimum foci size (0.3 × 0.3 × 4 µm) for quantification.

**Figure 5:**
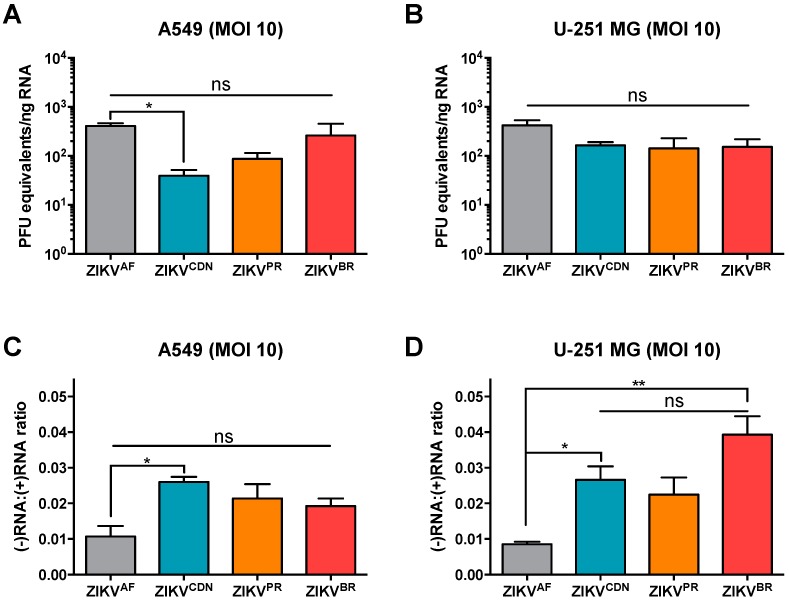
Asian lineage ZIKV isolates induce a higher ratio of negative:positive strand RNA. (**A**) A549 and (**B**) U-251 MG cells were infected with ZIKV at MOI = 10 and 24 h post-infection intracellular positive strand viral RNA was quantified by qRT-PCR. Data are normalized to GAPDH and expressed relative to a standard curve of PFU equivalents per ng input RNA. (**C**) A549 cells and (**D**) U-251 MG cells were infected with ZIKV at MOI = 10 and 24 h post-infection the relative amounts of positive and negative strand ZIKV genomes was quantified by qRT-PCR. Data are expressed as a ratio of negative:positive strand RNA. Values represent mean ± SEM of two or three independent experiments. Asterisks indicate significant differences: * *p* < 0.05, ** *p* < 0.01.

Finally, to further investigate whether the number and brightness of dsRNA foci was related to an increase in viral replication, we also quantified negative- and positive-strand viral RNA (vRNA) by qRT-PCR analyses (Figure 5). Given that three of the ZIKV isolates used in this study are patient isolates and therefore we are not able to determine absolute vRNA copy numbers using a standard curve of in vitro-transcribed RNA, we quantified positive-strand vRNA relative to a standard curve of vRNA from ZIKV stocks of known titer (log PFU equivalents) as has been done previously [8]. While ZIKV^CDN^ produced less positive-strand vRNA than ZIKV^AF^ in A549 cells, possibly due to the lower proportion of ZIKV^CDN^-infected cells (Figure 3A), there were no other statistically significant differences in intracellular positive-strand vRNA across ZIKV isolates in either cell type. This indicates that all of the ZIKV isolates produce similar levels of intracellular positive-strand vRNA after infection at high MOI.

Next, we wanted to determine the relative amount of the replicative intermediate, negative-strand vRNA (Figure 5C,D). Interestingly, on average ZIKV^AF^ produced approximately one copy of negative-strand intermediate vRNA for every 100 positive-strand vRNAs in both A549 and U-251 MG cells. In contrast, the Asian lineage isolates produced 1.9 to 2.6 negative strands for every 100 positive-strand vRNAs in A549 cells, and 2.2 to 3.9 negative-strands for every 100 positive strands in U-251 MG cells. This indicates that approximately 1.8 to 4.5-fold more negative-strand intermediate RNA is produced during infection with Asian lineage isolates when compared with ZIKV^AF^. Taken together, this data suggests that the Asian lineage isolates are able to produce more negative-strand intermediate vRNA than the African lineage isolate, and that the contemporary isolates, particularly ZIKV^BR^, induce more dsRNA in U-251 MG cells.

## 4. Discussion

The recent expansion of the previously obscure ZIKV beyond its historical endemic range in equatorial Africa and Southeast Asia has piqued scientific and public health interest in emerging viral diseases. Phylogenetic analyses and investigations into recently acquired genetic polymorphisms suggest that recent evolutionary changes may have contributed to the rapid emergence and novel neurological pathogenesis observed in contemporary ZIKV outbreaks in the Americas [1,18]. In this study, we selected four ZIKV isolates from different points in its phylogenetic history (Figure S1) and compared viral replicative fitness in human lung carcinoma (A549) and human astrocytoma (U-251 MG) cell lines. We compared two ZIKV isolates from the 2015–2016 outbreaks in Puerto Rico and Brazil (ZIKV^PR^ and ZIKV^BR^) to an earlier Asian lineage isolate (ZIKV^CDN^) and the historical Uganda 1947 reference isolate (ZIKV^AF^). We found that Asian lineage isolates produce more negative-strand intermediate vRNA, and that the contemporary ZIKV isolates, and in particular ZIKV^BR^, produce more dsRNA and significantly higher viral titers over a single infectious cycle (which was more pronounced in U-251 MG cells), although these contemporary isolates may be impaired in cell spread over multiple infectious cycles.

Several groups have shown strain-dependent differences in ZIKV infection in cell culture and mouse models of infection, albeit with conflicting results [38]. Our data suggests that strain-dependent differences in viral replicative fitness are highly dependent on both cell type and the MOI used, which may explain some of these discrepancies in the literature. For example, several groups have demonstrated enhanced infectivity and replication by contemporary American ZIKV isolates, similar to what we observed herein at high MOI [39,40]. In contrast, other groups have shown that African lineage ZIKV isolates display higher infectivity and increased cytopathicity in cell culture than Asian lineage isolates when low MOIs (≤1) were used, where few cells are predicted to be infected, similar to what we observed herein [41,42,43,44]. Although it is postulated that the highly-passaged African lineage isolate (MR766) may not accurately represent the phenotype of circulating African lineage ZIKV isolates, it should be noted that a recent study suggests that MR766 behaves similarly to other low-passage African lineage isolates in cell culture, and that all African lineage ZIKV isolates tested were more cytopathic than Asian lineage isolates at low MOIs [42]. The observed trend in relative CPEs is consistent with our flow cytometry data and the viral titers observed herein, suggesting that there is not an inherent difference in cytopathicity of the four ZIKV isolates, but rather CPE elicited is a consequence of active viral replication. Notably, we found that apoptosis does not appear to be the main driver of cell death during infection, with the exception of ZIKV^BR^, which induced substantially more cleaved caspase-3 positive cells in both cell types. Moreover, we found that ZIKV infection induced more apoptosis in A549 cells than the U-251 MG cells, which is consistent with a recent study that suggests that human fetal astrocytes are resistant to ZIKV-induced apoptosis [16]. However, we cannot rule out the possibility that apoptosis may be more prevalent at later time points post-infection, as a recent study suggests a higher proportion of activated caspase-3 positive cells at later time points in both A549 cells and human fetal astrocytes with proportions similar to what we report here at earlier time points [16].

In addition, our data suggests that contemporary ZIKV isolates may be impaired in cell spread when compared with the African and early Asian ZIKV isolates, resulting in lower CPE in cell culture when infected at a low MOI. Several factors may contribute to this reduced infectivity and our results do not exclude the possibility that reduced viral binding may account for the differences in cell spread. Correspondingly, a recent study suggests that the structural proteins of a Brazilian ZIKV isolate are impaired in their ability to initiate an infection [41]. Moreover, it is also possible that the increased viral replication induced by the contemporary isolates in the initial round of infection induces a more robust antiviral response in bystander cells, which then restricts viral spread. Further research into strain-dependent differences in induction of innate immune responses may thus help to elucidate mechanisms by which the antiviral response is able to restrict ZIKV infection and whether the contemporary isolates are altered in their abilities to counter host antiviral defenses.

Interestingly, we noticed that both the contemporary isolates induced significantly higher amounts of dsRNA in the U-251 MG astrocytoma cells, while ZIKV^BR^ specifically was able to replicate to significantly higher titers in these cells. Moreover, the contemporary isolates had a higher MFI of dsRNA-positive cells, with ZIKV^BR^ in particular having a greater number of dsRNA foci per cell in the astrocytomas. Further quantification of positive- and negative-strand vRNA during infection revealed that the Asian lineage isolates generate more negative-strand replicative intermediate RNA than the African lineage isolate. Overall, this data supports the hypothesis that Asian lineage isolates may have a higher replicative fitness in astrocytoma cells. This is supported by the greater ratio of negative-strand replicative intermediate vRNA, and greater amounts of dsRNA foci per cell, as well as the significant increase in titer observed in ZIKV^BR^ in this cell type (which correlates well with the increase in negative-strand vRNA and dsRNA foci). On the other hand, it could be argued that ZIKV^AF^ may have the replicative advantage since it produces similar levels of positive-strand vRNA from fewer negative-strand intermediates; however, this does not appear to translate into higher viral infectious titers for this isolate. In addition to the higher infectious titers observed in ZIKV^BR^ infection, it is also possible that, consistent with the greater amount of dsRNA-positive cells during ZIKV^PR^ infection, this isolate may also mirror the increase in titer observed at later time points post-infection. However, our data suggests that ZIKV^PR^ titers have plateaued by 24 h post-infection at MOI 10 in both cell types (Figure 1B,C). As such, ZIKV^BR^ may have an additional advantage over ZIKV^PR^ in a post-replication step of the viral life cycle. In addition to the kinetic advantage ZIKV^BR^ has over a single infectious cycle, this isolate also appears to have a viral replicative fitness advantage over the other ZIKV isolates at low MOI when fewer cells are infected. The similar or higher viral titers produced by ZIKV^BR^ at the low MOI implies more infectious particles are released per cell during infection with this isolate. However, the higher titers of ZIKV^BR^ observed in the multistep growth curve, even when the % infected cells are low, may be due to compounded differences in replication kinetics over several rounds of viral replication.

An increase in viral replicative fitness of contemporary ZIKV isolates in astrocyte-derived cell types may translate into more severe neurological pathogenesis over the course of an infection. A current perspective on the neurotropic potential of ZIKV suggests that infected macrophages could potentially carry the virus into the developing brain through a “Trojan-horse” mechanism [45]. Once the virus is in the brain, other investigations conducted in mice and primary human tissue samples suggest that ZIKV preferentially infects astrocytes [16,25,26]. In concordance with these studies, all of the ZIKV isolates examined herein were able to efficiently infect and replicate in human astrocytoma cells. This affinity for astrocytes is likely due to their high expression of AXL, a putative receptor for ZIKV, which interacts with Gas6 to promote ZIKV infection [46,47]. Thus, examining the differential affinity displayed by different isolates in binding to the AXL/Gas6 viral entry receptor(s) may help to further elucidate the mechanism by which the contemporary strains elicit increased infection kinetics.

Taken together, our data suggests that the contemporary American ZIKV isolates may have evolved mechanisms to increase viral replication and/or infectious particle production in astrocyte-derived cell types. Furthermore, our comparative analysis in human lung carcinoma (A549) and astrocytoma (U-251 MG) cells further highlights the importance of cell-intrinsic factors in studies of ZIKV replicative fitness. Although similar levels of positive-strand intracellular vRNA were observed across isolates, the Asian lineage isolates had substantially more negative-strand replicative intermediate vRNA when compared with the African lineage isolate, suggesting that the Asian lineage isolates may have a greater replicative fitness. The strain-specific differences in ZIKV replication were more pronounced in astrocytoma cells than in the lung carcinoma cells, and thus, the U-251 MG astrocytoma cells may serve as a more appropriate cell culture model for investigating isolate-dependent differences in ZIKV replicative fitness. However, it is important to note that with the exception of ZIKV^AF^, which was derived from an infectious cDNA, all other isolates were low passage patient isolates and, hence, we cannot rule out the possibility that they may harbor additional mutations resulting from adaptation to and/or propagation in cell culture. Thus, further investigations of these strain-specific polymorphisms that contribute to the observed differences in viral replicative fitness will be conducted by introduction of polymorphisms into infectious cDNAs that are now available for some of the isolates used herein [30,48]. Moreover, thorough investigation of cell-type-specific responses to infection may help elucidate the propensity for ZIKV to invade placental and neural tissues. Future studies must therefore consider the relationship between cell-intrinsic and strain-specific factors when examining ZIKV-host interactions.

## Supplementary Materials

The following are available online at http://www.mdpi.com/1999-4915/10/12/728/s1, **Table S1.** List of ZIKV Genbank accession numbers of sequences used in phylogenetic analysis, **Table S2.** Source host, isolation, and passage history of ZIKV strains used in this study, **Table S3.** Percent amino acid identity matrix of the ZIKV isolates used in this study, **Figure S1.** Phylogenetic analysis of ZIKV isolates, **Figure S2.** ZIKV isolates differentially induce apoptosis, **Figure S3.** ZIKV isolates differ in infectivity at low MOI, **File S1.** Amino acid sequence alignment of the ZIKV isolates used in this study.
