# Supplementary material for: Contemporary Zika Virus Isolates Induce More dsRNA and Produce More Negative-Strand Intermediate in Human Astrocytoma Cells
Source: Viruses. 2018 Dec 19;10(12):728. doi: 10.3390/v10120728 (PMC6316034; doi:10.3390/v10120728)
Supplement: Supplementary file 1 [file viruses-10-00728-s001.pdf]

Table S1. List of ZIKV Genbank accession numbers of sequences used in phylogenetic analysis.

Name	Genbank Accession #	Name	Genbank Accession #
Brazil/2015	KU321639	Honduras/2015	KX694534
Brazil/2015	KU527068	Honduras/2016	KX262887
Brazil/2015	KX280026	Malaysia/1966	KX377336
Brazil/2015	KX520666	Martinique/2015	KU647676
Brazil/2016	KU926310	Mexico/2015	KX247632
Brazil/2016	KU926309	Mexico/2016	KX446951
Cambodia/2010	KU955593	Micronesia/2007	EU545988
Canada/2013	KF993678	Nigeria/1968	KU963574
Central African Republic/1968	KF383115	Panama/2015	KX156775
Central African Republic/1976	KF268948	Panama/2015	KX198135
Central African Republic/1979	KF268950	Phillippines/2012	KU681082
Central African Republic/1980	KF268949	Puerto Rico/2015	KX377337
China/2016	KU761564	Samoa/2016	KX185891
Columbia/2015	KX548902	Senegal/1968	KF383116
Columbia/2016	KX247646	Senegal/1984	HQ234501
Dominican Republic/2016	KU853012	Senegal/1997	KF383117
Ecuador/2016	KX879603	Senegal/2001	KF383119
French Guiana/2015	KU758877	Singapore/2016	KX813683
French Polynesia/2013	KJ776791	Suriname/2015	KU312312
French Polynesia/2014	KX447511	Suriname/2016	KU937936
Guadeloupe/2016	KX673530	Thailand/2014	KU680181
Guatemala/2015	KU501217	Tonga/2016	KX806557
Guatemala/2016	KU870645	Uganda/1947	KU955594
Haiti/2014	KU509998	USA/2016	KX842449
Haiti/2016	KX051563	Venezuela/2016	KX702400

Table S2. Source host, isolation and passage history of ZIKV strains used in this study

Lineage	Strain	Source Host	Year	Location	Passage History	Genbank Accession
African	MR 766	Rhesus monkey	1947	Uganda	146x SM[a], 1x C6/36[b], 1x Vero[c]	HQ234498.1
Asian	PLCal_ZV	Human	2013	Canada/ Thailand	4x Vero	KF993678
Asian	PRVABC59	Human	2015	Puerto Rico	3x Vero	KU501215.1
Asian	HS-2015-BA-01	Human	2015	Brazil	3x C6/36, 1x Vero	KX520666

[a]SM = suckling mouse;
[b]C6/36 = *Aedes albopictus* cells;
[c]Vero = African green monkey kidney cells.

Table S3. Percent amino acid identity matrix of the ZIKV isolates used in this study.

	**Uganda/1947**	**Canada/2013**	**Puerto Rico/2015**	**Brazil/2015**
**Uganda/1947**	100	97.01	96.90	98.86
**Canada/2013**	97.01	100	99.76	99.62
**Puerto Rico/2015**	96.90	99.76	100	99.71
**Brazil/2015**	98.86	99.62	99.71	100

Figure S1

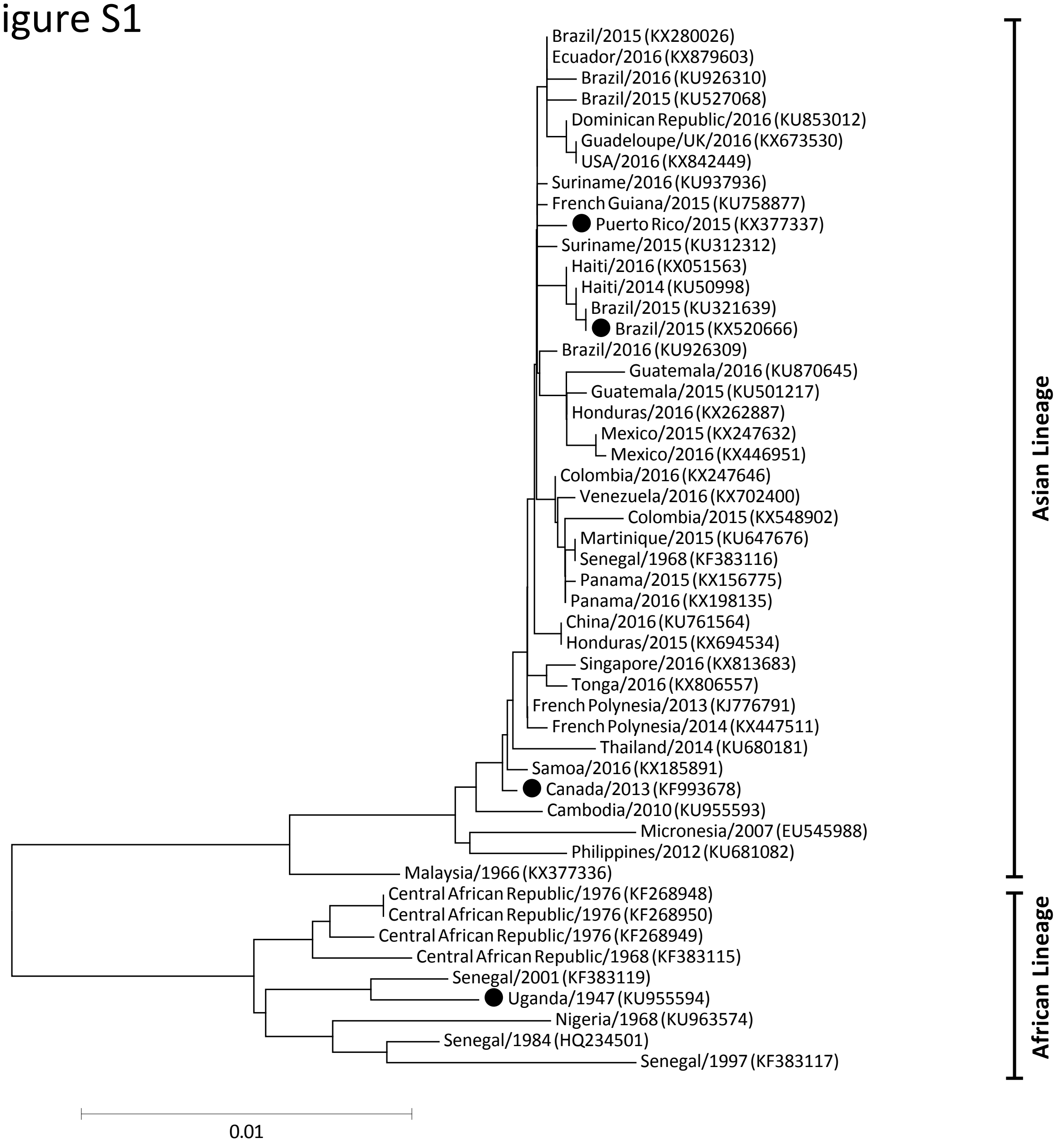

Figure S1. Phylogenetic analysis of selected ZIKV strains. Neighbor joining phylogenetic tree showing the position of the viral isolates used in this study (indicated by black circles) in the global ZIKV diversity. African and Asian lineages are indicated as well as country of origin, year of isolation, and GenBank accession number.

Figure S2

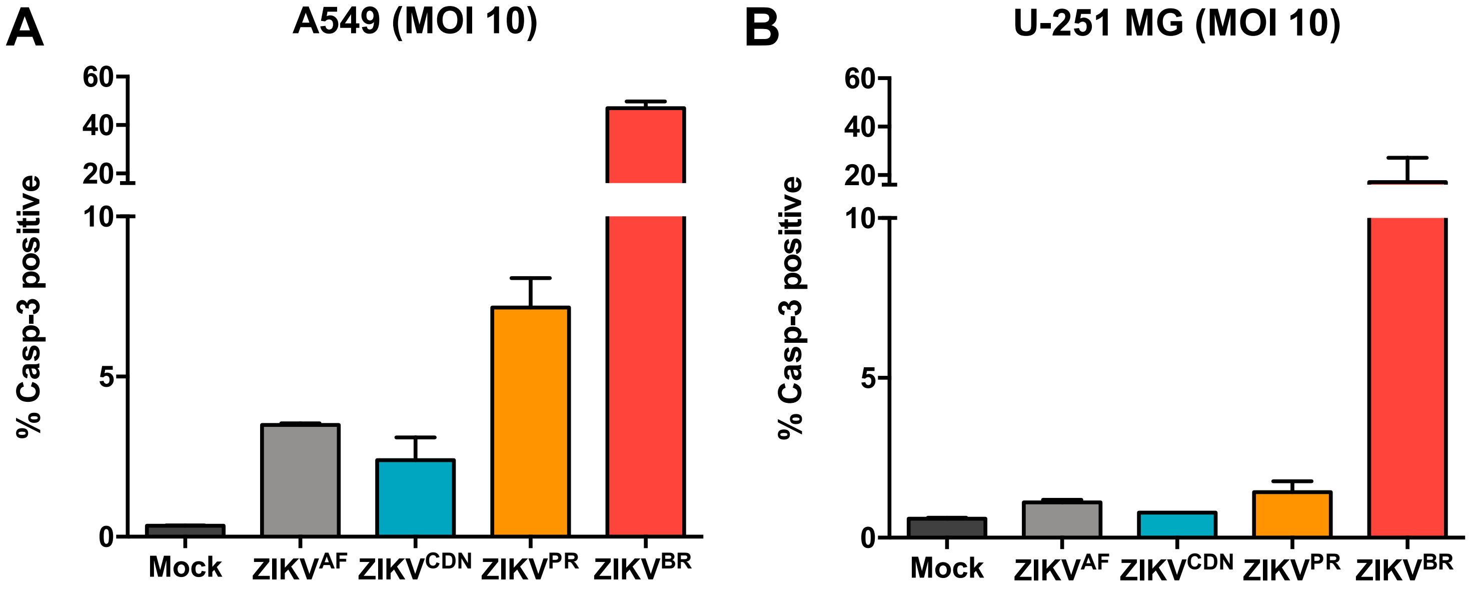

Figure S2. ZIKV isolates differentially induce apoptosis. (A) A549 cells and (B) U-251 MG cells were infected with ZIKV at MOI = 10 and 24 h post-infection the percentage of cells positive for cleaved caspase-3 was determined by flow cytometry. Values represent mean ± SD of two independent experiments.

Figure S3

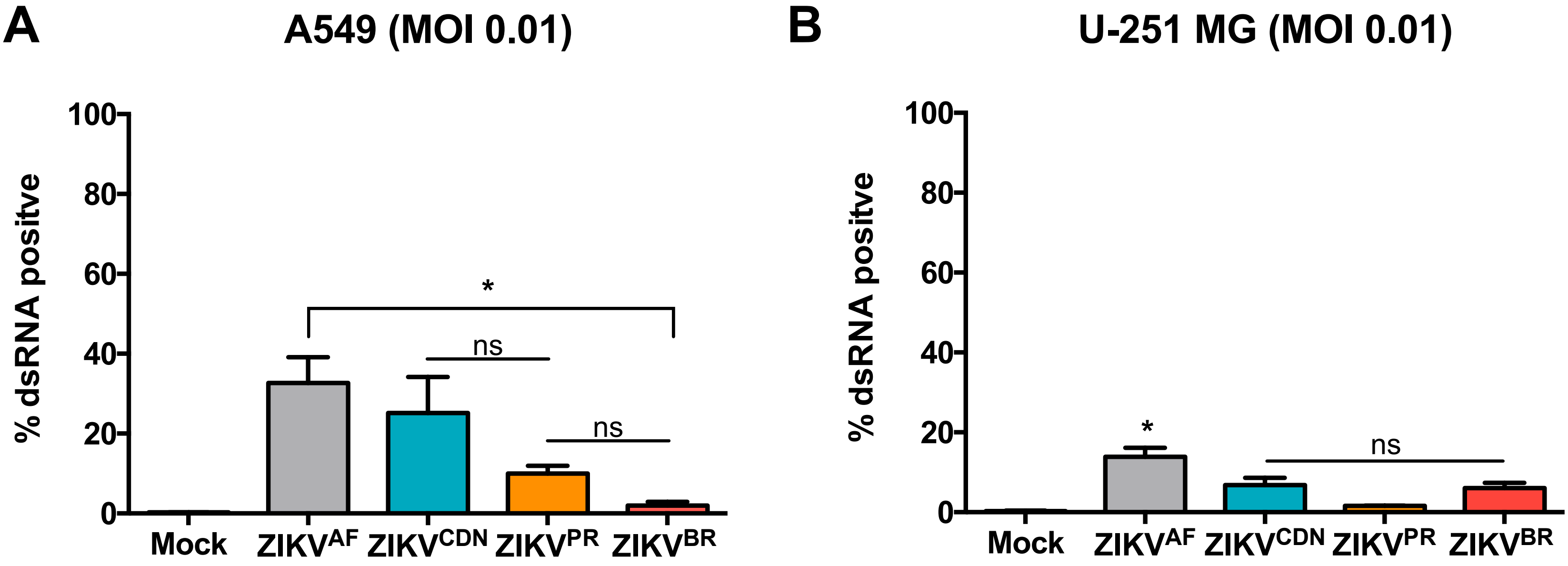

Figure S3. ZIKV isolates differ in infectivity at low MOI. (A) A549 cells and (B) U-251 MG cells were infected with ZIKV at MOI = 0.01 and 72 h post-infection the percentage of dsRNA-positive cells was determined by flow cytometry. Values represent mean ± SEM of at least three independent experiments. Asterisks indicate significant differences in % infected cells: ** $p < 0.01$, *** $p < 0.001$.

File S1: Amino acid sequence alignment of the ZIKV isolates used in this study.

```
                         Capsid
Uganda/1947         MKNPKKKSGGFRIVNMLKRGVARVNPLGGLKRLPAGLLLGHGPIRMVLAILAFLRFTAIK   60
Canada/2013         ························S·F·································   60
Puerto_Rico/2015    ························S·F·································   60
Brazil/2015         ························S·F·································   60

Uganda/1947         PSLGLINRWGSVGKKEAMEIIKKFKKDLAAMLRIINARKERKRRGADTSIGIIGLLLTTA   120
Canada/2013         ········································K····T···V··V·······   120
Puerto_Rico/2015    ···················T····················K········V··V·······   120
Brazil/2015         ········································K········V··V·······   120

                      pre M
Uganda/1947         MAAEITRRGSAYYMYLDRSDAGKAISFATTLGVNKCHVQIMDLGHMCDATMSYECPMLDE   180
Canada/2013         ····V·················E····P····M···YI······················   180
Puerto_Rico/2015    ····V·············N···E····P····M···YI······················   180
Brazil/2015         ····V·············N···E····P····M···YI······················   180

Uganda/1947         GVEPDDVDCWCNTTSTWVVYGTCHHKKGEARRSRRAVTLPSHSTRKLQTRSQTWLESREY   240
Canada/2013         ····························································   240
Puerto_Rico/2015    ····························································   240
Brazil/2015         ····························································   240
                                                                      E protein
Uganda/1947         TKHLIKVENWIFRNPGFALVAVAIAWLLGSSTSQKVIYLVMILLIAPAYSIRCIGVSNRD   300
Canada/2013         ·····R·············A·A······································   300
Puerto_Rico/2015    ·····R·············A·A······································   300
Brazil/2015         ·····R·············A·A······································   300

Uganda/1947         FVEGMSGGTWVDVVLEHGGCVTVMAQDKPTVDIELVTTTVSNMAEVRSYCYEASISDMAS   360
Canada/2013         ····························································   360
Puerto_Rico/2015    ····························································   360
Brazil/2015         ············I···············································   360

Uganda/1947         DSRCPTQGEAYLDKQSDTQYVCKRTLVDRGWGNGCGLFGKGSLVTCAKFTCSKKMTGKSI   420
Canada/2013         ·················································A··········   420
Puerto_Rico/2015    ·················································A··········   420
Brazil/2015         ·················································A··········   420

Uganda/1947         QPENLEYRIMLSVHGSQHSGMI----GYETDENRAKVEVTPNSPRAEATLGGFGSLGLDC   476
Canada/2013         ······················VNDT·H··········I·····················   480
Puerto_Rico/2015    ······················VNDT·H··········I·····················   480
Brazil/2015         ······················VNDT·H··········I·····················   480

Uganda/1947         EPRTGLDFSDLYYLTMNNKHWLVHKEWFHDIPLPWHAGADTGTPHWNNKEALVEFKDAHA   536
Canada/2013         ····························································   540
Puerto_Rico/2015    ····························································   540
Brazil/2015         ····························································   540

Uganda/1947         KRQTVVVLGSQEGAVHTALAGALEAEMDGAKGKLFSGHLKCRLKMDKLRLKGVSYSLCTA   596
Canada/2013         ································R·S·························   600
Puerto_Rico/2015    ································R·S·························   600
Brazil/2015         ································R·S·························   600

Uganda/1947         AFTFTKVPAETLHGTVTVEVQYAGTDGPCKIPVQMAVDMQTLTPVGRLITANPVITESTE   656
Canada/2013         ······I·······················V·A···························   660
Puerto_Rico/2015    ······I·······················V·A···························   660
Brazil/2015         ······I·······················V·A···························   660

Uganda/1947         NSKMMLELDPPFGDSYIVIGVGDKKITHHWHRSGSTIGKAFEATVRGAKRMAVLGDTAWD   716
Canada/2013         ······················E·····································   720
Puerto_Rico/2015    ······················E·····································   720
Brazil/2015         ······················E·····································   720

Uganda/1947         FGSVGGVFNSLGKGIHQIFGAAFKSLFGGMSWFSQILIGTLLVWLGLNTKNGSISLTCLA   776
Canada/2013         ······AL··································M·············M···   780
Puerto_Rico/2015    ······AL··································M·············M···   780
Brazil/2015         ······AL··································M·············M···   780
                                  NS1
Uganda/1947         LGGVMIFLSTAVSADVGCSVDFSKKETRCGTGVFIYNDVEAWRDRYKYHPDSPRRLAAAV   836
Canada/2013         ····L·····························V·························   840
Puerto_Rico/2015    ····L·····························V·························   840
Brazil/2015         ····L·····························V·························   840
```

```
Uganda/1947         KQAWEEGICGISSVSRMENIMWKSVEGELNAILEENGVQLTVVVGSVKNPMWRGPQRLPV    896
Canada/2013         ·····D················R·····································    900
Puerto_Rico/2015    ·····D················R·····························G········    900
Brazil/2015         ·····D················R·····································    900

Uganda/1947         PVNELPHGWKAWGKSYFVRAAKTNNSFVVDGDTLKECPLEHRAWNSFLVEDHGFGVFHTS    956
Canada/2013         ·······································K····················    960
Puerto_Rico/2015    ·······································K····················    960
Brazil/2015         ···············H·······················K····················    960

Uganda/1947         VWLKVREDYSLECDPAVIGTAVKGREAAHSDLGYWIESEKNDTWRLKRAHLIEMKTCEWP    1016
Canada/2013         ························K··V································    1020
Puerto_Rico/2015    ························K··V································    1020
Brazil/2015         ························K··V································    1020

Uganda/1947         KSHTLWTDGVEESDLIIPKSLAGPLSHHNTREGYRTQVKGPWHSEELEIRFEECPGTKVY    1076
Canada/2013         ·········I···························M·····················H    1080
Puerto_Rico/2015    ·········I···························M·····················H    1080
Brazil/2015         ·········I···························M·····················H    1080

Uganda/1947         VEETCGTRGPSLRSTTASGRVIEEWCCRECTMPPLSFRAKDGCWYGMEIRPRKEPESNLV    1136
Canada/2013         ············································································    1140
Puerto_Rico/2015    ············································································    1140
Brazil/2015         ············································································    1140

                          NS2A
Uganda/1947         RSMVTAGSTDHMDHFSLGVLVILLMVQEGLKKRMTTKIIMSTSMAVLVVMILGGFSMSDL    1196
Canada/2013         ·······································I········A···········    1200
Puerto_Rico/2015    ·······································I········A···········    1200
Brazil/2015         ·······································I········A···········    1200

Uganda/1947         AKLVILMGATFAEMNTGGDVAHLALVAAFKVRPALLVSFIFRANWTPRESMLLALASCLL    1256
Canada/2013         ···A·····················I··································    1260
Puerto_Rico/2015    ···A·····················I··································    1260
Brazil/2015         ···A·····················I··································    1260

Uganda/1947         QTAISALEGDLMVLINGFALAWLAIRAMAVPRTDNIALPILAALTPLARGTLLVAWRAGL    1316
Canada/2013         ····························V·······T·A·····················    1320
Puerto_Rico/2015    ····························V·······T·A·····················    1320
Brazil/2015         ····························V·······T·A·····················    1320

                                                                         NS2B
Uganda/1947         ATCGGIMLLSLKGKGSVKKNLPFVMALGLTAVRVVDPINVVGLLLLTRSGKRSWPPSEVL    1376
Canada/2013         ·····F···························L··························    1380
Puerto_Rico/2015    ·····F···························L··························    1380
Brazil/2015         ·····F···························L··························    1380

Uganda/1947         TAVGLICALAGGFAKADIEMAGPMAAVGLLIVSYVVSGKSVDMYIERAGDITWEKDAEVT    1436
Canada/2013         ············································································    1440
Puerto_Rico/2015    ············································································    1440
Brazil/2015         ············································································    1440

Uganda/1947         GNSPRLDVALDESGDFSLVEEDGPPMREIILKVVLMAICGMNPIAIPFAAGAWYVYVKTG    1496
Canada/2013         ····················D···············T·······················    1500
Puerto_Rico/2015    ····················D···············T·······················    1500
Brazil/2015         ····················D···············T·······················    1500

                      NS3
Uganda/1947         KRSGALWDVPAPKEVKKGETTDGVYRVMTRRLLGSTQVGVGVMQEGVFHTMWHVTKGAAL    1556
Canada/2013         ·························································S··    1560
Puerto_Rico/2015    ·························································S··    1560
Brazil/2015         ·························································S··    1560

Uganda/1947         RSGEGRLDPYWGDVKQDLVSYCGPWKLDAAWDGLSEVQLLAVPPGERARNIQTLPGIFKT    1616
Canada/2013         ·································H··························    1620
Puerto_Rico/2015    ·································H··························    1620
Brazil/2015         ·································H··························    1620

Uganda/1947         KDGDIGAVALDYPAGTSGSPILDKCGRVIGLYGNGVVIKNGSYVSAITQGKREEETPVEC    1676
Canada/2013         ··················································R·········    1680
Puerto_Rico/2015    ··················································R·········    1680
Brazil/2015         ··················································R·········    1680
```

```
Uganda/1947         FEPSMLKKKQLTVLDLHPGAGKTRRVLPEIVREAIKKRLRTVILAPTRVVAAEMEEALRG    1736
Canada/2013         ....................................T.......................    1740
Puerto_Rico/2015    ....................................T.......................    1740
Brazil/2015         ....................................T.......................    1740

Uganda/1947         LPVRYMTTAVNVTHSGTEIVDLMCHATFTSRLLQPIRVPNYNLYIMDEAHFTDPSSIAAR    1796
Canada/2013         ............................................................    1800
Puerto_Rico/2015    ............................................................    1800
Brazil/2015         ............................................................    1800

Uganda/1947         GYISTRVEMGEAAAIFMTATPPGTRDAFPDSNSPIMDTEVEVPERAWSSGFDWVTDHSGK    1856
Canada/2013         ............................................................    1860
Puerto_Rico/2015    ............................................................    1860
Brazil/2015         ........................................................Y...    1860

Uganda/1947         TVWFVPSVRNGNEIAACLTKAGKRVIQLSRKTFETEFQKTKNQEWDFVITTDISEMGANF    1916
Canada/2013         .........................................H......V...........    1920
Puerto_Rico/2015    .........................................H......V...........    1920
Brazil/2015         .........................................H......V...........    1920

Uganda/1947         KADRVIDSRRCLKPVILDGERVILAGPMPVTHASAAQRRGRIGRNPNKPGDEYMYGGGCA    1976
Canada/2013         .....................................................L......    1980
Puerto_Rico/2015    .....................................................L......    1980
Brazil/2015         .....................................................L......    1980

Uganda/1947         ETDEGHAHWLEARMLLDNIYLQDGLIASLYRPEADKVAAIEGEFKLRTEQRKTFVELMKR    2036
Canada/2013         ....D.......................................................    2040
Puerto_Rico/2015    ....D.......................................................    2040
Brazil/2015         ....D.......................................................    2040

Uganda/1947         GDLPVWLAYQVASAGITYTDRRWCFDGTTNNTIMEDSVPAEVWTKYGEKRVLKPRWMDAR    2096
Canada/2013         ............................................R...............    2100
Puerto_Rico/2015    ............................................RH..............    2100
Brazil/2015         ............................................RH..............    2100
                                       NS4A
Uganda/1947         VCSDHAALKSFKEFAAGKRGAALGVMEALGTLPGHMTERFQEAIDNLAVLMRAETGSRPY    2156
Canada/2013         ......................F.....................................    2160
Puerto_Rico/2015    ......................F.....................................    2160
Brazil/2015         ......................F.....................................    2160

Uganda/1947         KAAAAQLPETLETIMLLGLLGTVSLGIFFVLMRNKGIGKMGFGMVTLGASAWLMWLSEIE    2216
Canada/2013         ............................................................    2220
Puerto_Rico/2015    ............................................................    2220
Brazil/2015         ............................................................    2220
                                              protein 2K             NS4B
Uganda/1947         PARIACVLIVVFLLLVVLIPEPEKQRSPQDNQMAIIIMVAVGLLGLITANELGWLERTKN    2276
Canada/2013         ...........................................................S    2280
Puerto_Rico/2015    ...........................................................S    2280
Brazil/2015         ...........................................................S    2280

Uganda/1947         DIAHLMGRREEGATMGFSMDIDLRPASAWAIYAALTTLITPAVQHAVTTSYNNYSLMAMA    2336
Canada/2013         .LS...........I......................F......................    2340
Puerto_Rico/2015    .LS...........I......................F......................    2340
Brazil/2015         .LS..................................F......................    2340

Uganda/1947         TQAGVLFGMGKGMPFYAWDLGVPLLMMGCYSQLTPLTLIVAIILLVAHYMYLIPGLQAAA    2396
Canada/2013         ...................F......I.................................    2400
Puerto_Rico/2015    ...................F......I.................................    2400
Brazil/2015         ...................F......I.................................    2400

Uganda/1947         ARAAQKRTAAGIMKNPVVDGIVVTDIDTMTIDPQVEKKMGQVLLIAVAISSAVLLRTAWG    2456
Canada/2013         ................................................V...I.S.....    2460
Puerto_Rico/2015    ................................................V...I.S.....    2460
Brazil/2015         .............................................M..V...I.S.....    2460

Uganda/1947         WGEAGALITAATSTLWEGSPNKYWNSSTATSLCNIFRGSYLAGASLIYTVTRNAGLVKRR    2516
Canada/2013         ............................................................    2520
Puerto_Rico/2015    ............................................................    2520
Brazil/2015         ............................................................    2520
```

```
                    ↱NS5
Uganda/1947         GGGTGETLGEKWKARLNQMSALEFYSYKKSGITEVCREEARRALKDGVATGGHAVSRGSA   2576
Canada/2013         ············································································   2580
Puerto_Rico/2015    ············································································   2580
Brazil/2015         ············································································   2580

Uganda/1947         KLRWLVERGYLQPYGKVVDLGCGRGGWSYYAATIRKVQEVRGYTKGGPGHEEPMLVQSYG   2636
Canada/2013         ·················I······················K···················   2640
Puerto_Rico/2015    ·················I············V·········K············V······   2640
Brazil/2015         ·················I······················K············V······   2640

Uganda/1947         WNIVRLKSGVDVFHMAAEPCDTLLCDIGESSSSPEVEETRTLRVLSMVGDWLEKRPGAFC   2696
Canada/2013         ······································A·····················   2700
Puerto_Rico/2015    ······································A·····················   2700
Brazil/2015         ······································A·····················   2700

Uganda/1947         IKVLCPYTSTMMETMERLQRRHGGGLVRVPLSRNSTHEMYWVSGAKSNIIKSVSTTSQLL   2756
Canada/2013         ··············L······Y··························T···········   2760
Puerto_Rico/2015    ··············L······Y··························T···········   2760
Brazil/2015         ··············L······Y··························T···········   2760

Uganda/1947         LGRMDGPRRPVKYEEDVNLGSGTRAVASCAEAPNMKIIGRRIERIRNEHAETWFLDENHP   2816
Canada/2013         ··························V············N······S·······F·····   2820
Puerto_Rico/2015    ··························V············N······S·······F·····   2820
Brazil/2015         ··························V············N······S·······F·····   2820

Uganda/1947         YRTWAYHGSYEAPTQGSASSLVNGVVRLLSKPWDVVTGVTGIAMTDTTPYGQQRVFKEKV   2876
Canada/2013         ·····················I······································   2880
Puerto_Rico/2015    ·····················I······································   2880
Brazil/2015         ·····················I······································   2880

Uganda/1947         DTRVPDPQEGTRQVMNIVSSWLWKELGKRKRPRVCTKEEFINKVRSNAALGAIFEEEKEW   2936
Canada/2013         ·············I·SM···········H·······························   2940
Puerto_Rico/2015    ···············SM···········H·······························   2940
Brazil/2015         ···············SM···········H·······························   2940

Uganda/1947         KTAVEAVNDPRFWALVDREREHHLRGECHSCVYNMMGKREKKQGEFGKAKGSRAIWYMWL   2996
Canada/2013         ·················K··········Q·······························   3000
Puerto_Rico/2015    ·················K··········Q·······························   3000
Brazil/2015         ·················K··········Q·······························   3000

Uganda/1947         GARFLEFEALGFLNEDHWMGRENSGGGVEGLGLQRLGYILEEMNRAPGGKMYADDTAGWD   3056
Canada/2013         ······································V····S·I···R··········   3060
Puerto_Rico/2015    ······································V····S·I···R··········   3060
Brazil/2015         ······································V····S·I···R··········   3060

Uganda/1947         TRISKFDLENEALITNQMEEGHRTLALAVIKYTYQNKVVKVLRPAEGGKTVMDIISRQDQ   3116
Canada/2013         ····R··············K···A····I·················K·············   3120
Puerto_Rico/2015    ····R··············K···A····I·················K·············   3120
Brazil/2015         ····R··············K···A····I·················K·············   3120

Uganda/1947         RGSGQVVTYALNTFTNLVVQLIRNMEAEEVLEMQDLWLLRKPEKVTRWLQSNGWDRLKRM   3176
Canada/2013         ········································RS····N·············   3180
Puerto_Rico/2015    ········································RS····N·············   3180
Brazil/2015         ········································RS····N·············   3180

Uganda/1947         AVSGDDCVVKPIDDRFAHALRFLNDMGKVRKDTQEWKPSTGWSNWEEVPFCSHHFNKLYL   3236
Canada/2013         ··········································D···············H·   3240
Puerto_Rico/2015    ··········································D···············H·   3240
Brazil/2015         ··········································D···············H·   3240

Uganda/1947         KDGRSIVVPCRHQDELIGRARVSPGAGWSIRETACLAKSYAQMWQLLYFHRRDLRLMANA   3296
Canada/2013         ············································································   3300
Puerto_Rico/2015    ············································································   3300
Brazil/2015         ············································································   3300

Uganda/1947         ICSAVPVDWVPTGRTTWSIHGKGEWMTTEDMLMVWNRVWIEENDHMEDKTPVTKWTDIPY   3356
Canada/2013         ···S····························V···························   3360
Puerto_Rico/2015    ···S····························V···························   3360
Brazil/2015         ···S····························V···························   3360
```

```
Uganda/1947          LGKREDLWCGSLIGHRPRTTWAENIKDTVNMVRRIIGDEEKYMDYLSTQVRYLGEEGSTP    3416
Canada/2013          ··························N·································    3420
Puerto_Rico/2015     ··························N·································    3420
Brazil/2015          ··························N·································    3420

Uganda/1947          GVL    3419
Canada/2013          ···    3423
Puerto_Rico/2015     ···    3423
Brazil/2015          ···    3423
```
